# Ultrasonic-Assisted Water-Rich Natural Deep Eutectic Solvents for Sustainable Polyphenol Extraction from Seaweed: A Case Study on Cultivated *Saccharina latissima*

**DOI:** 10.1021/acssuschemeng.4c06736

**Published:** 2024-09-26

**Authors:** Liaqat Zeb, Anne Sophie Gerhardt, Benjamin Alexander Johannesen, Jarl Underhaug, Monica Jordheim

**Affiliations:** Department of Chemistry, University of Bergen, Bergen 5007, Norway

**Keywords:** seaweed, water-rich NADES, polyphenols, qNMR, marine sustainability, blue bioeconomy

## Abstract

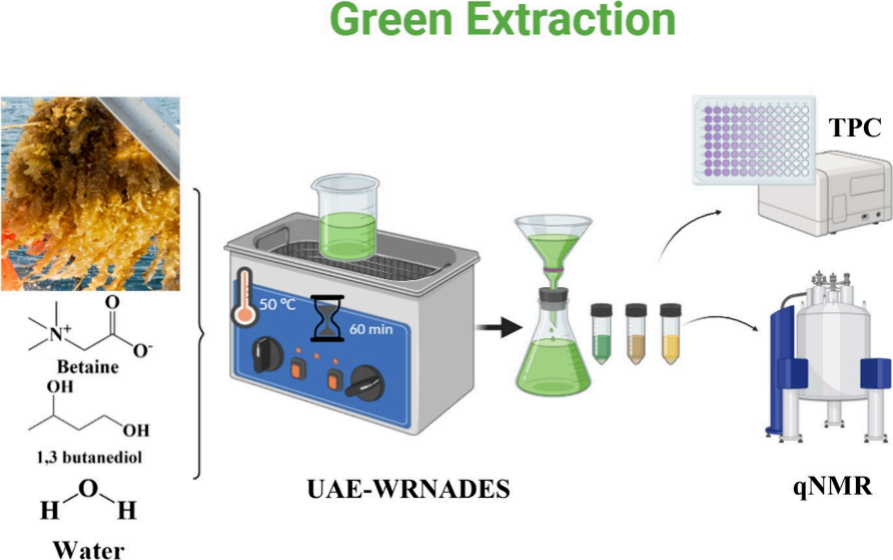

This case study introduces a green, 1 h single-step method using water-rich natural deep eutectic solvent (WRNADES) for ultrasound-assisted extraction (UAE) of polyphenols from*Saccharina latissima*, a commercially cultivated brown seaweed. The extraction efficiency was evaluated using a selective quantitative NMR method (s-qNMR) and the traditional nonselective colorimetric total phenolic content assay (TPC). Initial 6 h extractions in traditional solvents (methanol, ethanol, acetone, and ethyl acetate) showed a 40–60% increase in polyphenolic yields in 50% aqueous solutions measured by the TPC method. Six different water-rich (50%) NADES (WRNADES) combinations were tested (choline chloride/betaine with lactic acid, citric acid, and 1,3-butanediol), with betaine and 1,3-butanediol (1:1) proving most effective. Parameters for the WRNADES were optimized using Box–Behnken design response surface methodology, resulting in a 1:20 w/w biomass to solvent ratio and a 1 h extraction time at 50 °C. The WRNADES extraction process was refined into a scalable, single-step procedure and compared with traditional solvent extractions (6 h, 50% aqueous methanol and acetone). A final XAD-7 polyphenol recovery step was included in all extractions. The optimized WRNADES extraction yielded 15.97 mg GAE/g of the dry weight recovered polyphenolic extract (s-qNMR), exceeding the 6 h 50% aqueous methanol (12.4 mg GAE/g) and acetone (11.4 mg GAE/g) extractions. Thus, the UAE-WRNADES method presented in this case study provides a cost-effective, sustainable, and eco-friendly alternative for the extraction of phenolic compounds from seaweed. It promotes the development of environmentally friendly production processes within the seaweed biorefinery.

## Abstract 2

This study demonstrates an eco-friendly, single-step, ultrasound-assisted extraction using water-rich natural deep eutectic solvent to sustainably extract polyphenols from seaweed.

## 1 Introduction

In recent years, the interest in natural products from the marine environment has increased, partly driven by the blue bioeconomy and the need for the discovery of new sustainable bioresources. Since the overall environmental impacts of seaweed farming remain relatively low compared to other seafood and biomass sources,^1^ there is an emerging industry based on seaweed cultivation.^2,3^ Macroalgae, or seaweeds, have diverse chemical profiles of valuable compounds such as alginates, fucoidan, laminarin, cellulose, mannitol, polyphenols, and pigments.^4,5^ These marine compounds find applications across various industries including food, feed, cosmetics, and pharmaceuticals.^5,6^ In particular, the hunt for natural antioxidants derived from seaweed is on the rise, given their broad benefits and potential for diverse industrial applications.^7,8^ Phenolics, or polyphenols, are among the high-value antioxidant products of interest from marine sources, and they are also known for their diverse bioactivities related to antimicrobial, anticancer, antifungal, antiviral, and anti-inflammatory properties.^9−18^ However, while terrestrial plant phenolics have been extensively studied, research on seaweed phenolics remains limited.^5,7,8,19^ An important element in this knowledge gap is related to challenges in the efficient and green extraction of polyphenols from seaweed. Extraction of polyphenols from macro algae traditionally involves organic solvents such as ethanol, acetone, and methanol.^8,20−23^ However, these methods have drawbacks including prolonged extraction times, high solvent consumption, low yields, toxicity, volatility, environmental concerns, and potential explosive characteristics.^8,21,24,25^ An alternative approach involves the use of natural deep eutectic solvents (NADESs), which offer environmentally friendly alternatives due to their low or nontoxic nature, biodegradability, and reusability properties.^21,24,25^

NADESs represent the next generation of solvents, composed of a hydrogen bond acceptor (HBA) and a hydrogen bond donor (HBD).^24,26^ Their renewable and biodegradable nature, which combines vegetal cellular constituents like choline derivatives, sugars, amino acids, organic acids, or polyols, makes them ideal for the sustainable utilization of biomasses.^25^ NADESs transform into viscous, transparent, and homogeneous solvents through stirring and heating processes.^26^ They offer advantages, such as negligible toxicity, low volatility, a broad polarity range, adjustable viscosity, and miscibility, with water. They can also be used as an antifreezing agent for natural products.^27,28^ Given these diverse characteristics, NADESs have emerged as green solvents for extracting metabolites from plants and algae.^26^ Additionally, ultrasound-assisted extraction (UAE) NADES has previously also been effectively employed to enhance the extraction of natural products from various biomasses, offering a more efficient and environmentally friendly alternative to traditional extraction methods.^21,29^

In this case study, water-rich natural deep eutectic solvents (WRNADESs) were used in a green approach to extract phenolic compounds from a Norwegian commercially cultivated brown seaweed, *Saccharina latissima (S. latissima)*. The extraction parameters were optimized by Box-Behnken design response surface methodology (BBD-RSM). Ultrasonic-assisted extraction (UAE) was used to improve the extraction efficiency of phenolic compounds in the WRNADES.^21,29^ The UAE-WRNADES was utilized in a streamlined process that encompassed a single-step extraction. Subsequently, its performance was compared to that of organic solvents. Finally, its eco-scale greenness was evaluated and contrasted with traditional extraction methods. To address accurate measurement of the total polyphenolic content, an advanced selective quantitative NMR (s-qNMR) method was used for the evaluation of extraction yields together with a traditional colorimetric assay (TPC).^30,31^ s-qNMR enables sample recovery, direct measurement, and structural insights with high selectivity, specificity, and reproducibility, offering molecular-level accuracy and precise polyphenol quantification, which surpass the traditional Folin–Ciocalteu (FC) TPC method in specificity and resistance to interference. This green extraction approach aligns with the principles of green chemistry, reducing the environmental burden of solvents compared with conventional extraction methods.

## 2 Materials and Methods

### 2.1 Chemicals

The chemicals and solvents used in the experiments were of analytical grade. The following chemicals were procured from Sigma-Aldrich (St. Louis, MO, USA): betaine, choline chloride, lactic acid, citric acid, 1,3-butanediol, FC reagent, gallic acid, methanol, ethanol, acetone, ethyl acetate, DMSO-*d*_6_ (with a TMS content of 0.03%), and acetic acid (with a purity of ≥95–99%). The deionized water used was from the University of Bergen, Norway (Milli-Q, IQ 7010). All other reference standards used in the study were procured from Merck.

### 2.2 Seaweed Biomass—*S. latissima*

*S. latissima* biomass was collected by Lerøy Ocean Forest in May 2023 at the Trollsøy location, Bergen, Norway during their annual harvest. The fresh *S. latissima* biomass was washed in distilled water, air-dried in the lab, kept away from direct sun exposure, and then chopped into small pieces using a blender. Both fresh and dry biomasses were stored in the refrigerator at −20 °C for further experimental use.

### 2.3 WRNADES Preparation

The WRNADES were prepared by adopting the heating methodology as a simple and low-energy consumption method.^21,29^ In WRNADES preparation, a known molar ratio of the HBD, HBA, and water was mixed to form homogeneous and transparent eutectic solvents (Table 1). WRNADES were prepared at 50–60 °C (for 1 h stirring on a hot plate) by mixing choline chloride (Ch) and betaine (B) with 50% (w/w) of water and different HBD: lactic acid (Ch-LA, B-LA), citric acid (Ch-CA, B-CA), 1,3-butanediol (Ch-BD and B-BD) (Table 1). The WRNADES were stored in closed flasks at room temperature for further experiment analysis.

**Table 1:** Composition of the Water-Rich Natural Deep Eutectic Solvent (WRNADES), the Hydrogen Bond Acceptor (HBA) and Hydrogen Bond Donor (HBD), Their Molar Ratios, Water Content (%), and Abbreviations^a^

WRNADES	HBA	HBD	molar ratio	water (%)	abbreviation
1	choline chloride	lactic acid	(3:1)	50	Ch-LA
2	choline chloride	citric acid	(1:1)	50	Ch-CA
3	choline chloride	1,3-butanediol	(1:1)	50	Ch-BD
4	betaine	lactic acid	(3:1)	50	B-LA
5	betaine	citric acid	(1:1)	50	B-CA
6	betaine	1,3-butanediol	(1:1)	50	B-BD

a Water % was based on the molar ratio weight.

### 2.4 Six-Hour Extraction with Organic Solvents

Organic solvents methanol, ethanol, acetone, and ethyl acetate were used to extract phenolic compounds from the *S. latissima* dry biomass. The 50% aqueous and 100% pure organic solvents of 40 mL were used for extraction of 1 g dry biomass (1:40) in a 100 mL tube/flask and kept vertexing at 400 rpm for 6 h. Ethyl acetate and water were partially immiscible in a 50% aqueous solution, the experiment was conducted under continuous agitation at 400 rpm. This agitation facilitated effective contact between the immiscible phases, enabling the extraction of polyphenols. After vertexing, the mixture was filtered or centrifuged at 5000 rpm to separate the supernatant. The samples were washed with 15 mL of distilled water with respective organic solvents to recover the extract completely. All organic solvents, including 50% aqueous acetone, were evaporated using a rotary evaporator (BUCHI Rotavapor R-100, Green VAC PC 3001 VARIO) to recover the final product/extract.

### 2.5 Six-Hour Extraction with WRNADES

The prepared 50% aqueous WRNADES (Table 1 and Section 2.3) were used to extract polyphenols from 1 g of *S. latissima* dry biomass (1:20 w/w), mixed, and vortexed at 400 rpm for 6 h. To ensure the complete recovery of the extract, the samples were washed with 20 mL of distilled water and filtered WRNADES extract. The mixture was filtered or centrifuged at 5000 rpm to separate the supernatant.

### 2.6 Optimization of Extraction Parameters for UAE-WRNADES by BBD-RSM

A 60% water-rich betaine-1,3-butanediol (1:1) WRNADES was integrated with an UAE (KS-500DE, 500w, 22.5 L) approach, and the parameters were optimized by BBD-RSM to evaluate significant parameters and optimize the process. Three significant variables were studied (Table 2), temperature (°C) (A), sample-to-solvent ratio/*S. latissima* biomass-to-WRNADES ratio (DM, w/w) (B), and extraction time (min) (C), including one response; the total phenolic content measured (TPC). The temperature was carefully controlled and maintained at 51 °C to seamlessly integrate the UAE-NADES process into a single step while preventing the degradation of polyphenols. In total, 15 sets of extraction experiments were performed and 3 replicates at the central points. After the experiments, the samples were filtered or centrifuged at 5000 rpm for 8 min, and the supernatant was collected. The samples were washed with distilled water and filtered WRNADES extract, and the TPC of the extracts was determined by the FC method. Statistical comparisons were performed with Design Expert and Origin ANOVA.

**Table 2:** Box–Behnken Design (BBD) Variables for UAE-WRNADES Extraction of Polyphenols from *Saccharina latissima*: Symbols and Value Ranges

variables	symbols	minimum value	maximum value
temperature (°C)	A	43	51
sample to solvent ratio (w/w)	B	10	20
extraction time (min)	C	20	100

### 2.7 Single-Step 1 h Extraction of UAE-WRNADES

The optimized parameters of the UAE-WRNADES extraction of phenolic compounds from *S. latissima* were further integrated into a single-step extraction and then increased into multiple biomasses. Briefly, the NADES components of betaine-1,3-butanediol (1:1), 60% water, and *S. latissima* dry biomass were directly added to a tube/flask and continued treatment for 60 min at 50 °C. The extraction process was conducted from 1 to 3 g of *S. latissima* dry biomass with the increasing of the same extraction parameters as UAE-WRNADES extraction.

### 2.8 TPCs Analysis by FC Colorimetric Method

The FC colorimetric method as described by^21,29^ was used with modification for the TPC determination. In this method, an alkaline medium and Folin’s reagents promote the reduction of molybdates and tungstates. The blue chromogen phenolate anions formed with a maximum absorbance of 760 nm. A standard calibration curve of gallic acid was used as the phenolic standard. The linear range of gallic acid was 4–64 μg mL^–1^ with a coefficient of determination of 0.9999 and *y* = 0.059*x* + 0.0169. The method was adopted to a 96-well microplate; first, 100 μL of extracts diluted up to 1000 μL in distilled water were mixed with 100 μL of Folin’s reagent for 6–8 min. To alkalinize the medium, 150 μL of a sodium carbonate solution (20%) was added to the samples and kept in the dark for 30 min. When the samples were prepared, a UV–vis spectrophotometer (Biotek Eon SN.259245) measured the absorbance at 760 nm. The blanks used for the WRNADES extracts were diluted at the same concentration. For each organic solvent, the same solvent was used as the blank. The TPC amount of extract was calculated using gallic acid equivalent (mg of GAE) per mg of the extract used. The experiments were repeated in triplicate.

TPC assay was performed for dry weight yield (after XAD-7 adsorbent) according to an optimized method for brown seaweeds using the FC reagent.^30^ The method involves using 0.2 mL of the sample, blank, or standard combined with 1.59 mL of FC reagent and 4.0 mL of 20% (w/v) sodium carbonate (Na_2_CO_3_). The mixture is then diluted to a total volume of 20 mL with MQ water. The samples were then left in the dark for an incubation period of 30 min. Following this, the absorbance was recorded at 760 nm using a Biochrom Libra S32 UV instrument (Biochrom Cambridge, United Kingdom). Gallic acid was used as a standard for the TPC method’s linearity, sensitivity, precision, and accuracy. Each standard or sample is analyzed three times to ensure the data are statistically significant.

### 2.9 Recovery of Phenolic Compounds

Dry biomass (20 mg) was used to extract polyphenols with 50% aqueous methanol, acetone, and 60% aqueous WRNADES. For methanol and acetone, the extracts were recovered using a rotary evaporator, while the water in WRNADES was removed under reduced pressure using a rotary evaporator. Polyphenols in crude extracts of 50% aqueous methanol, acetone, and 60% aqueous WRNADES were recovered by an Amberlite XAD-7 adsorbent-filled column with a 2.6 cm inside diameter to give a bed height of 20 cm. The XAD-7 adsorbent was pretreated with 300 mL of ethanol and washed with 300 mL of MQ water before loading the methanol, acetone, and WRNADES phenolic extracts. After loading the WRNADES extract into the XAD-7 adsorbent column, the column was thoroughly washed with 600 mL of Milli-Q water to remove NADES, sugars, and salts. The phenolic compounds captured by the resin were then eluted with 600 mL of 100% ethanol. The methanol and acetone extracts were washed similarly to the WRNADES extract and washed with methanol. The solvents were evaporated using a rotary evaporator under vacuum pressure at 30 °C. The samples were dried under nitrogen and then freeze-dried for 24 h. The final phenolic contents were weighted and kept at −20 °C for further TPC and s-qNMR analysis.

### 2.10 TPC Quantification by s-qNMR Analysis

1D ^1^H qNMR was used in combination with 2D NMR data (^13^C-HSQC, ^13^C-HMBC, and ^15^N-HSQC) in order to perform a selective quantification of the TPC (s-qNMR). Prior to NMR analysis, the samples were filtered through a Whatman Puradisc 25 nylon membrane filter (pore size: 0.45 μm; diameter: 25 mm). Then, 1 mL of each sample was dried under nitrogen, freeze-dried, and dissolved in 0.75 mL DMSO-*d*_6_ (0.03% TMS) with DMSO_2_ as the internal standard (*C* = 10 mM) and transferred to 5 mm NMR tubes. All NMR spectra were acquired at 298 K using a 600 MHz Bruker AVANCE NEO spectrometer equipped with a QCI-P Cryoprobe (Bruker BioSpin, Zürich, Switzerland).

1D ^1^H qNMR was used for the integration of individual aromatic signals within a selected region (7.0–5.5 ppm).^30^ The spectra were acquired using the *zg* pulse sequence, with 128 scans, 2 dummy scans, an acquisition time of 4 s, and a relaxation delay (*d*_1_) of 10 s. The delay between each scan (relaxation delay + acquisition time) was ≥5*T*_1_, ensuring ≥99% accuracy for the quantification.

^13^C-HSQC spectra were obtained using the *hsqcedetgpsisp2.3* pulse sequence, with 292 scans, 32 dummy scans, a ^1^H sweep width of 13.02 ppm, and a ^13^C sweep width of 200.0 ppm, and the ^13^C-HMBC spectra were obtained using a *hmbcetgpl3nd* pulse sequence with nonuniform sampling (50%), 192 scans and 16 dummy scans, *d*_1_ of 2 s, ^1^H sweep width 13.02 ppm, and ^13^C sweep width 220.0 ppm. In addition, a ^15^N-HSQC spectrum was acquired to deselect ^1^H attached to nitrogen, which is not part of the polyphenolic content. The nitrogen spectra were acquired using a *hsqcetgp* pulse sequence with 50% nonuniform sampling. The number of scans was 128 with 16 dummy scans, a ^1^H sweep width of 15.15 ppm, and a ^15^N sweep width of 200.0 ppm. All polyphenolic quantifications were performed based on the following equation with DMSO_2_ as the internal standard (*C* = 10 mM, *n* = 6):
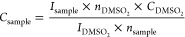
where *C* is the concentration in molar, *n* is the number of protons yielding the signal, and *I* is the signal integral.

## 3 Results and Discussion

### 3.1 Six-Hour Extraction of Polyphenols from *S. latissima* by Traditional Organic Solvents

**Figure 1:**
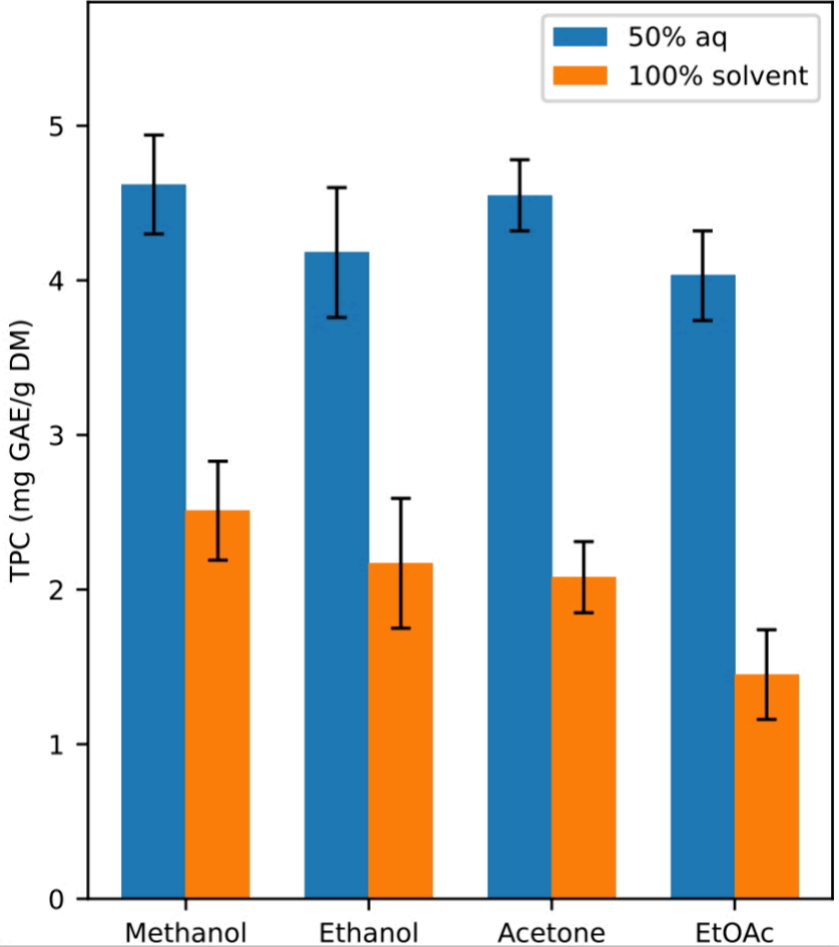
Extraction of polyphenols from *Saccharina latissima* with a traditional 6 h extraction method (1:40, biomass:solvent ratio) using 50% aqueous (blue) and 100% pure (orange) organic solvents of methanol, ethanol, acetone, and ethyl acetate (EtOAc). Quantification was performed using the total phenolic content (TPC) assay, and data were reported as milligram gallic acid (GAE) equivalents per gram dry weight of biomass.

Organic solvents, such as methanol, ethanol, acetone, and ethyl acetate, are used for the extraction of polyphenols from brown seaweeds,^5,8,20−22,24,32−34^ and different ratios of aqueous organic solvents have been tested with increased phenolic extraction yields.^5,35,36^ Therefore, the current traditional organic solvent extraction methods chosen for *S. latissima* were two series of four organic solvents (methanol, ethanol, acetone, ethyl acetate); one series based on 50% aqueous solvents and the other series based on pure solvents (Figure 1). All extractions were performed with 6 h of treatment time, and the phenolic yields were measured with the colorimetric TPC method for preliminary analysis.^30^

The major difference observed between the two series was the increased phenolic yields seen for the 50% aqueous solvents (Figure 1) compared to the pure solvents. This is according to previous findings.^5,34^ However, the differences observed in our study with *S. latissima* biomass are substantial, with a 40–60% observed increase in yields. Methanol and acetone showed slightly higher phenolic amounts extracted compared to the ethanol and ethyl acetate, measured with the nonselective TPC method. Considering the selectivity of the two solvent extraction series, besides from the phenolic content, the aqueous solvents were orange and the pure organic solvents were green, most likely attributed to increased amounts of carotenoids and chlorophylls extracted, respectively.

### 3.2 Six-Hour Extraction of Polyphenols from *S. latissima* by WRNADES

**Figure 2:**
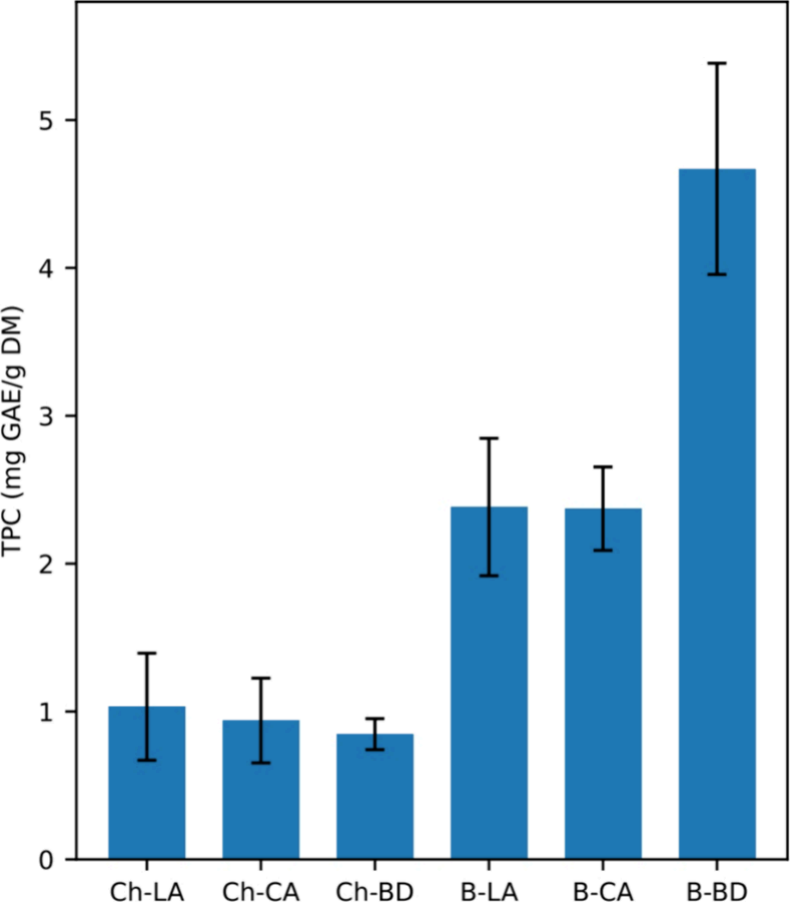
Polyphenols from *Saccharina latissima* extracted using six 50% aqueous WRNADES combinations in a 6 h method (Table 1). Quantified using the TPC assay, data reported as milligrams of GAE equivalents/gram of dry biomass.

To evaluate a more environmentally friendly extraction method, the selected seaweed *S. latissima* underwent extraction processes involving mixtures of choline chloride- and betaine-based NADESs. In addition, these processes used increased quantities of water, resulting in WRNADES, water-rich NADES. The choice of preparing water-rich NADES is based on the increased extraction efficacy of polyphenols from seaweeds observed for aqueous organic solvents (Section 3.1). Six different WRNADES were prepared (choline chloride:lactic acid; 3:1, choline chloride:citric acid; 1:1, choline chloride:1,3-butanediol; 1:1, betaine:lactic acid; 3:1, betaine:citric acid; 1:1, and betaine:1,3-butanediol; 1:1) (Table 1). The solvent-to-mass ratio and extraction time were maintained within a maximum and minimum range (Figure 2). Based on the traditional extraction methods, the extraction time was kept at 6 h and 50% percentage of water to assess the impact as a green strategy. The choline chloride WRNADES showed in general substantial lower polyphenolic extraction yields than the betaine-based WRNADES (Figure 2), with the highest yield seen for the betaine and 1,3-butanediol mixture (1:1) (TPC: 4.67 mg GAE/g DM). These results using *S. lattissima* are different compared to previous NADES extractions on other brown seaweeds as *Fucus vesiculosus* and*Ascophyllum nodosum*,^21,22^ where aqueous solutions containing choline chloride seemed to have higher potential for extracting polyphenols. However, the FC method (TPC) has been shown to particularly overestimate these species growing in more shallow waters, compared to the sublittoral species *S. latissima* and *Laminaria hyperborea*.^30^ For *F. vesiculosus* the FC method has been shown to overestimate by as much as ∼30%.^30^

### 3.3 Optimization of UAE-WRNADES by BBD-RSM

**Figure 3:**
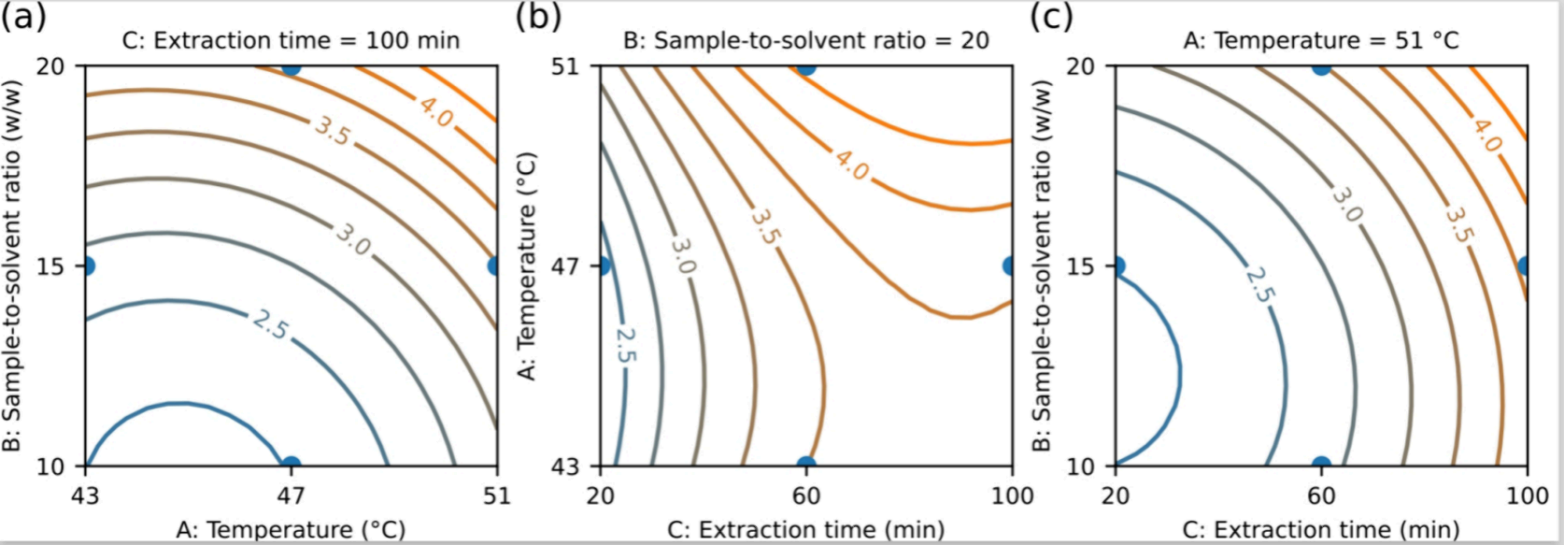
Contour plot of Box–Behnken design for UAE-WRNADES shows the different effect on the total phenolic content (TPC); (a) temperature (43–51 °C, *A*), sample-to-solvent ratio (10–20 w/w, *B*), and extraction time (100 min, *C*); (b) extraction time (20–100 min, *C*), temperature (43–51 °C, *A*), and sample-to-solvent ratio (20 w/w, *B*); (c) extraction time (20–100 min, *C*), sample-to-solvent ratio (10–20 w/w, *B*), and temperature (51 °C, *A*).

The betaine-based WRNADES with 1,3-butanediol (1:1) and a higher percent of water (60% water) were chosen based on our preliminary results to optimize the extraction parameters for *S. latissima*. The decision of increasing the water percentage from 50 to 60% was based on the higher solubility of phlorotannins in aqueous (70–80%) solvents.^37,38^ In addition, UAE was applied to facilitate the extraction process. Temperature (A), sample-to-solvent ratio (B), and extraction time (C) were evaluated. Tables 2 and 3 show the experimental conditions for the UAE-WRNADES with 15 runs, also including the response evaluated by the TPC results. Each parameter was set within the maximum and minimum range. The extracted polyphenol amounts varied from 1.91 to 4.67 mg of GAE/g of DM of *S. latissima* biomass. The ideal optimized parameters for extraction were 1:20 w/w biomass to solvent ratio, extraction time 60 min, and temperature 51 °C facilitated by UAE. In general, the amounts of polyphenols extracted increased with increasing solvent-to-biomass ratio, followed by extraction time and temperature (Table 3). The Box-Behnken contour plots (Figure 3) demonstrate the relationship between polyphenol amounts (TPC) in the *S. latissima* extract and the experimental variables (A–C). The verification experiments were conducted twice under specific conditions to validate the accuracy of the expected response values (Table S1). This discrepancy reaffirmed the adequacy of the applied design in establishing the optimized processing conditions for achieving the desired results.

**Table 3:** Box–Behnken Design (BBD) with Conditions and Experimentally Obtained Total Phenolic Content (TPC) Values^a^

		factor 1	factor 2	factor 3	response
std	run	*A*	*B*	*C*	TPC
5	1	43	15	20	1.98
7	2	43	15	100	2.65
13	3	47	15	60	2.79
6	4	51	15	20	2.48
10	5	47	20	20	2.25
9	6	47	10	20	1.91
11	7	47	10	100	2.41
14	8	47	15	60	2.76
8	9	51	15	100	3.39
2	10	51	10	60	3.09
12	11	47	20	100	3.78
1	12	43	10	60	2.44
15	13	47	15	60	2.69
4	14	51	20	60	4.45
3	15	43	20	60	3.55

a Variables are (A) temperature, (B) sample-to-solvent ratio, (C) extraction time, and their effect against a TPC. The data were reported as milligram gallic acid (GAE) equivalents per gram dry weight of biomass.

#### 3.3.1 Model Adjustment and Analysis of Variance

Table S1 presents the ANOVA results for the quadratic Box–Behnken design model applied to the extraction of phenolic contents from *S. latissima*. The table includes coded parameters, sum of squares, degrees of freedom (df), mean square, *F*-value, and *p*-value. The regression data analyses revealed a correlation between TPC and the experimental variables as per eq 1 (*p* = 0.0007).
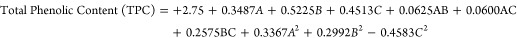
1

The predictive equation’s validity was confirmed through an *F*-test and analysis of variance (ANOVA). A satisfactory fit of the model to experimental data is indicated when derived from the ANOVA table and tabulated values, respectively. The ANOVA results, as depicted in Table S1, present the significance of the TPC model at *P* < 0.0007. These closely aligned parameters confirm a strong agreement between experimental and predicted values, indicating their applicability in prediction and optimization stages. Eq 1 illustrates the significance of all three selected independent variables affecting TPC extraction from *S. latissima*, displaying linear influences on TPC. This emphasizes the necessity for a comprehensive understanding of this response. Temperature (*A*), sample-to-solvent ratio (*B*), and extraction time (*C*) demonstrated significant linear impacts on the TPC extracted. Figure S1 shows the Pareto chart of the statistically analyzed data. Among the three parameters, the sample-to-solvent ratio (*B*) was the most important factor affecting the TPC, followed by extraction time (*C*) and temperature (*A*). The model was significant and the lack of fit was nonsignificant showing the fit of the model in Table S1.

### 3.4 Streamlined Single-Step 1 h Extraction of the UAE-WRNADES Method

The advanced extraction method, described in the preceding sections, was further refined into a streamlined single-step extraction process. This process utilizes the optimized WRNADES conditions and Box–Behnken design (BBD) parameters (*A*, 51 °C; *B*, 1:20 w/w biomass to solvent ratio of 60% aq betaine:1,3-butanediol (1:1); *C*, extraction time of 60 min) in a unified one-step extraction. After the UAE-WRNADES was optimized using BBD-RSM, the temperature was set at 50 °C for further experiments. This integrated single-step process eliminates the need for multiple stages during extraction, such as the initial preparation of NADES followed by their use in extracting phenolic compounds. As a result, this method significantly reduces both energy consumption and processing time.

The single-step method was then further conducted from 1 to 3 g of *S. latissima* biomass, maintaining the same extraction parameters. The extracted polyphenol amounts (mg GAE/g DW biomass) were consistent for the examined series of 1 g (4.44 ± 0.19 mg/g), 1.5 g (4.42 ± 0.25 mg/g), 2 g (4.40 ± 0.15 mg/g), 2.5 g (4.40 ± 0.25 mg/g), and 3 g (4.40 ± 0.15 mg/g), measured by the TPC assay. The consistent results demonstrate the effectiveness of this advanced extraction technique.

### 3.5 Comparison of Traditional Organic Solvent with UAE-WRNADES Extraction Using qNMR and TPC

To evaluate the yield of polyphenols from traditional extraction methods in comparison to our refined WRNADES method with improved selectivity, we utilized a novel selective qNMR method (s-qNMR) in addition to a nonselective one (qNMR), as described by Wekre et al.^30^ The s-qNMR method involves the exclusion of nonphenolic signals and signals from the same spin system in the 1D 1H spectrum using 2D NMR analysis prior to integration, improving selectivity and avoiding overestimation. The results were compared with those obtained by the conventional nonselective FC method (TPC).^30,31,39−41^

**Figure 4:**
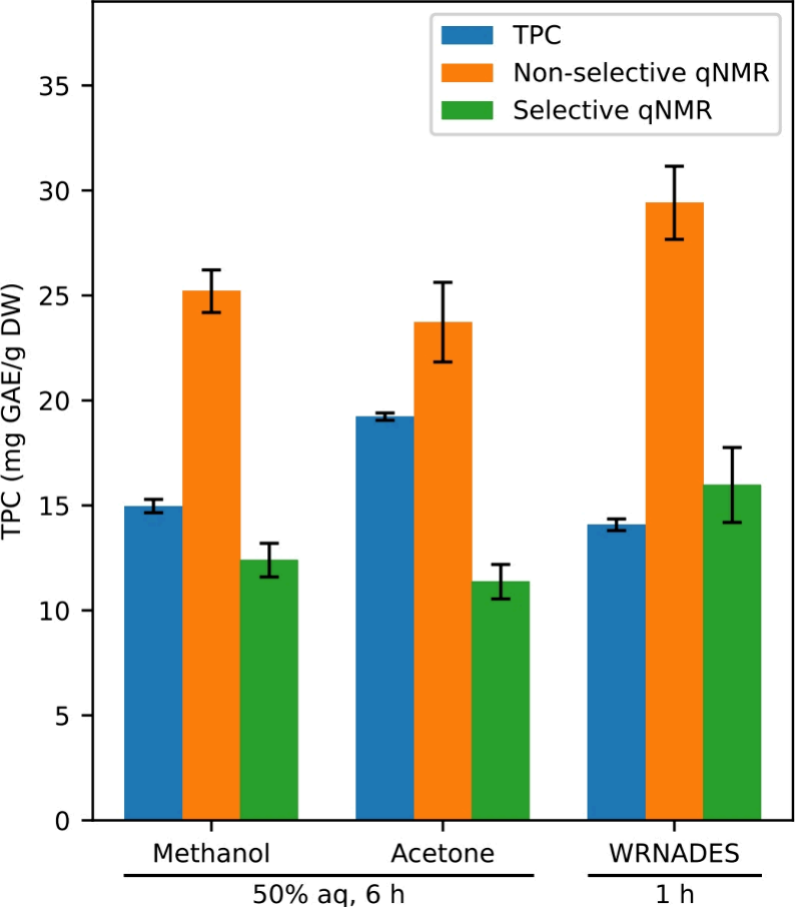
Polyphenolic content of *Saccharina latissima* was extracted with traditional (6 h, 1:40 ratio, 50% methanol/acetone) and optimized (1 h, 1:20 ratio, 60% WRNADES) methods. XAD-7 recovery step was included before analysis. Quantification was done with TPC assay (blue), qNMR (orange), and s-qNMR (green), and reported as mg GAE/g dry weight of recovered extract.

Quantification showed good coherence between the TPC and s-qNMR in all extracts (Figure 4). Our findings indicate thus that polyphenols, when extracted from *S. latissima* using WRNADES, appear to retain their properties and exhibit behavior similar to that of those extracted with traditional solvents. Additionally, it appears adequate to use the TPC method during NADES optimization for polyphenol quantification for the species used in this case study. As far as we are aware, no prior research has quantified polyphenols extracted from seaweeds using UAE-WRNADES through a s-q NMR method at the molecular level, nor has any study employed a water-rich strategy for polyphenol recovery or used a single-step extraction process. The current data are also in accordance with our previous observations, where brown seaweeds growing in sublittoral zones shows minor deviations between the two quantification methods (TPC and s-qNMR), this contrasts with what we see for the seaweed species in more shallow waters as *Fucus* and *Ascophylium.*(30) One additional observation is the tendency of TPC to overestimate the polyphenolic amounts when acetone is used as an extraction solvent. This phenomenon can be ascribed to the ability of acetone to extract a higher number of compounds that interfere with the TPC measurement compared to the more polyphenol-selective methanol and WRNADES.

### 3.6 Eco-Scale Methodology Assessment of Organic Solvent and UAE-WRNADES

The eco-scale method, introduced by Gałuszka et al., evaluates improvements in extraction processes for a more environmentally friendly approach by assigning penalty points to various process aspects.^42^ It comprises three zones: inappropriate green methods (0–50, red region), appropriate green methods (50–75, yellow region), and excellent green methods (75–100, green region). These penalty points assess energy consumption, reagents, instruments, waste production, and occupational hazards (gases and vapors).^29^Table 4 illustrates the comparison between methanol, acetone, and WRNADES. The UAE-WRNADES- based extraction holds a higher Eco-Scale score (98) than methanol (91) and acetone extraction (89). Table 4 suggests that UAE-WRNADES extraction improves greenness by 7 points from methanol and 9 points from acetone. Methanol and acetone are highly flammable solvents with toxicity and accrue lower points on the eco-scale, posing risks to safety and the environment. Hence, optimizing the UAE-WRNADES extraction of phenolic compounds from *S. latissima* increased the eco-scale score (Table 4), utilizing nonflammable solvents, ensuring a low cost with higher water content, eco-friendly, sustainable, nontoxic, biocompatible, and green chemistry-based process. WRNADES, a blend of natural substances like betaine, 1,3-butanediol, and a higher volume of water, offers a green alternative to traditional organic solvents used for the extraction of phenolic contents.

**Table 4:**
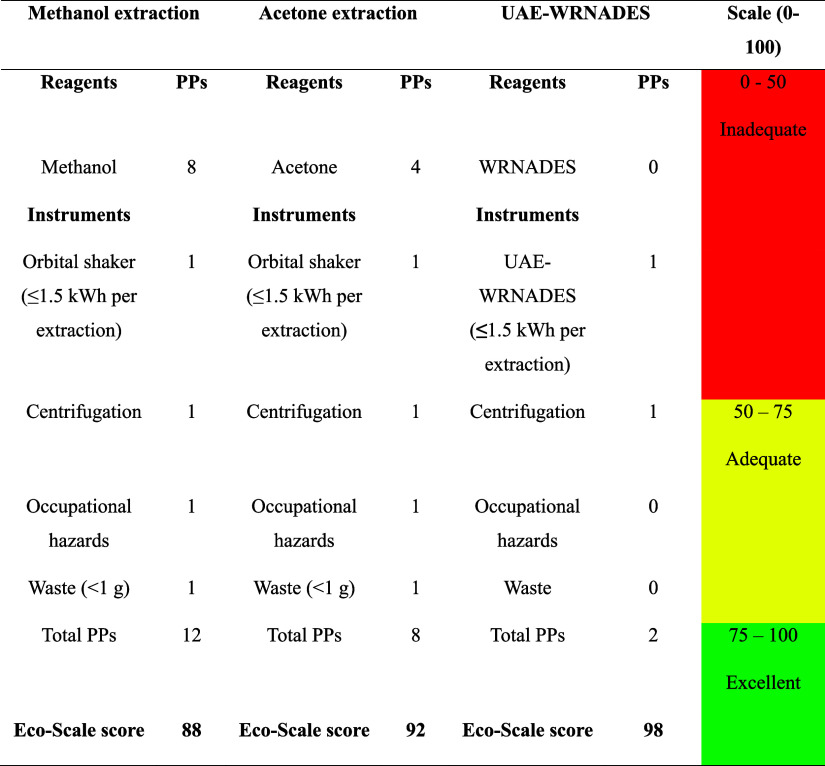
Eco-Scale Assessment of 50% Aqueous Methanol, Acetone, and 60% Aqueous UAE-WRNADES for Polyphenolic Content Extraction from *Saccharina latissima*^a^

a Comparison was based on the reagents, instruments, penalty points (PPs), and eco-scale score.

## 4 Conclusions

Seaweed serves as a sustainable source of polyphenolic compounds that have potential applications in the food, feed, and health sectors. However, the extraction of these polyphenols typically involves the use of organic solvents, which are not suitable for these industries or ideal from a cleaner production perspective. The current case study evaluated WRNADESs with UAE for extracting polyphenols from commercially cultivated *S. latissima*. A 1 h betaine and 1,3-butanediol (1:1) WRNADES showed an improved extraction efficiency of 15.97 mg GAE/g of dry weight extract, compared to a 6 h traditional 50% aqueous methanol (12.39 mg GAE/g) and acetone (11.36 mg GAE/g) extraction, evaluated by a selective qNMR method and a colorimetric FC assay (TPC). The extraction method using UAE-WRNADES was streamlined into a single step. This was achieved by directly incorporating NADES components, water, and dry biomass of *S. latissima* in one step.

This case study enhances our understanding of the use of water-rich NADES as a sustainable and cost-effective method for extracting polyphenols from seaweed. Importantly, it includes the application of a selective qNMR method for quantifying polyphenols within the NADES. This approach is novel and increases the reliability of our assessment of the extraction process with accurate determination at the molecular level. In general, it highlights the potential of using WRNADES to promote eco-friendly production processes in seaweed biorefinery.

## Data Availability

All data from this study are available in the article, with further details provided upon request to the corresponding author.

## Supporting Information Available

The Pareto chart derived from the Box–Behnken design (BBD) results of UAE-WRNADES for *Saccharina latissima*; ANOVA for BBD quadratic model of the UAE-WRNADES of the three variables temperature, sample-to-solvent ratio, extraction time, and their effect against a response total phenolic content (TPC) in *Saccharina latissima* (PDF)
