# Supplementary material for: Ultrasonic-Assisted Water-Rich Natural Deep Eutectic Solvents for Sustainable Polyphenol Extraction from Seaweed: A Case Study on Cultivated Saccharina latissima
Source: ACS Sustain Chem Eng. 2024 Sep 26;12(40):14921–9. doi: 10.1021/acssuschemeng.4c06736 (PMC11462601; doi:10.1021/acssuschemeng.4c06736)
Supplement: Supplementary file 1 — sc4c06736_si_001.pdf [file sc4c06736_si_001.pdf]

# Supporting Information

# Ultrasonic-Assisted Water-Rich Natural Deep Eutectic Solvents for Sustainable Polyphenol Extraction from Seaweed: A Case Study on Cultivated *Saccharina latissima*

Liaqat Zeb[a*], Anne Sophie Gerhardt[a,b], Benjamin Alexander Johannesen[a], Jarl Underhaug[a], Monica Jordheim[a*]

[a]Department of Chemistry, University of Bergen, Norway.

[b]Current address: Department of Safety, Chemistry and Biomedical laboratory sciences, Western University of Applied Science, Bergen, Norway

Corresponding author, Email: Monica.Jordheim@uib.no, Liaqat.Zeb@uib.no

Supporting information content:

**Number of pages: 3**

**Number of figures: 1**

**Number of tables: 1**

**Number of schemes: 0**

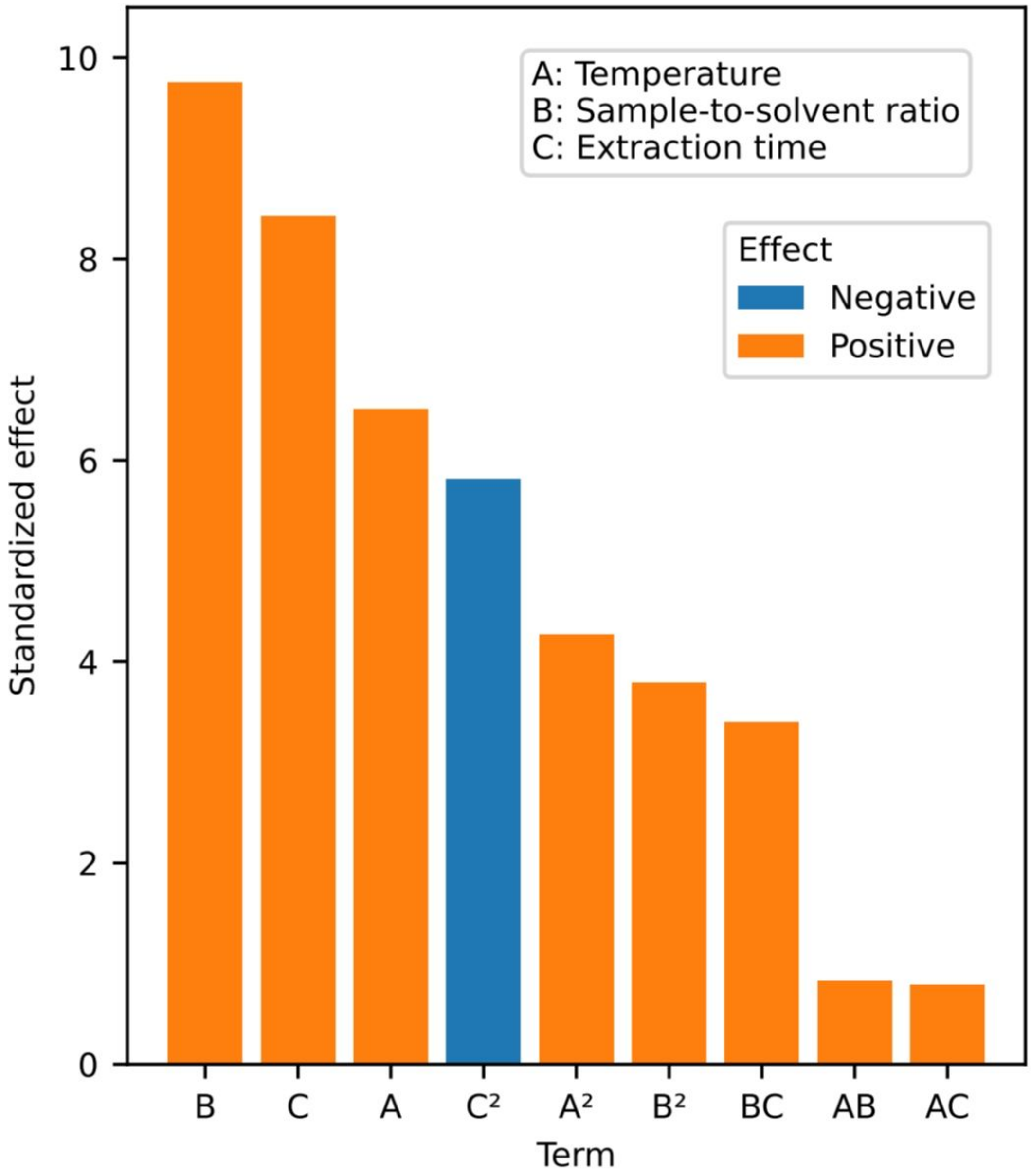


**SFigure 1**

The Pareto chart was derived from the Box-Behnken design results of UAE-WRNADES for *Saccharina latissima*. This chart illustrates the quadratic effects of three variables: temperature (A), sample-to-solvent ratio (B), and extraction time (C), as well as their interactions with each other. The sample-to-solvent ratio highly influenced the total phenolic content, followed by extraction time and temperature.

**STable 1** The analysis of variance for Box-Behnken Design (BBD) quadratic model of the UAE-WRNADES of the three variables (A) temperature, (B) sample-to-solvent ratio, (C) extraction time and their effect against a response total phenolic content (TPC) in *Saccharina latissima*. The table illustrates a list of sources, sum of squares, df, mean square, F-value, and *p*-value.

| Source | Sum of Squares | df | Mean Square | F-value | *p*-value | |
|---|---|---|---|---|---|---|
| Model | 6.72 | 9 | 0.7472 | 32.54 | 0.0007 | significant |
| A | 0.9730 | 1 | 0.9730 | 42.38 | 0.0013 | |
| B | 2.18 | 1 | 2.18 | 95.13 | 0.0002 | |
| C | 1.63 | 1 | 1.63 | 70.96 | 0.0004 | |
| AB | 0.0156 | 1 | 0.0156 | 0.6806 | 0.4469 | |
| AC | 0.0144 | 1 | 0.0144 | 0.6272 | 0.4643 | |
| BC | 0.2652 | 1 | 0.2652 | 11.55 | 0.0193 | |
| $A^2$ | 0.4185 | 1 | 0.4185 | 18.23 | 0.0079 | |
| $B^2$ | 0.3305 | 1 | 0.3305 | 14.39 | 0.0127 | |
| $C^2$ | 0.7756 | 1 | 0.7756 | 33.78 | 0.0021 | |
| Residual | 0.1148 | 5 | 0.0230 | | | |
| Lack of Fit | 0.1095 | 3 | 0.0365 | 13.86 | 0.0680 | not significant |
| Pure Error | 0.0053 | 2 | 0.0026 | | | |
| Cor Total | 6.84 | 14 | | | | |

*p*-values less than 0.0500 indicate that the model terms are significant. In this case A, B, C, BC, $A^2$, $B^2$, $C^2$ are significant model terms.
